# Supplementary material for: BLOS1 mediates kinesin switch during endosomal recycling of LDL receptor
Source: eLife. 2020 Nov 12;9:e58069. doi: 10.7554/eLife.58069 (PMC7688313; doi:10.7554/eLife.58069)
Supplement: Figure 1—source data 1. — Representative figure of CBB staining result of control and cKO mouse plasma proteins separated by SDS-PAGE. Arrow indicates the protein band which was further verified as apoE. [file elife-58069-fig1-data1.pptx]

## Slide 1
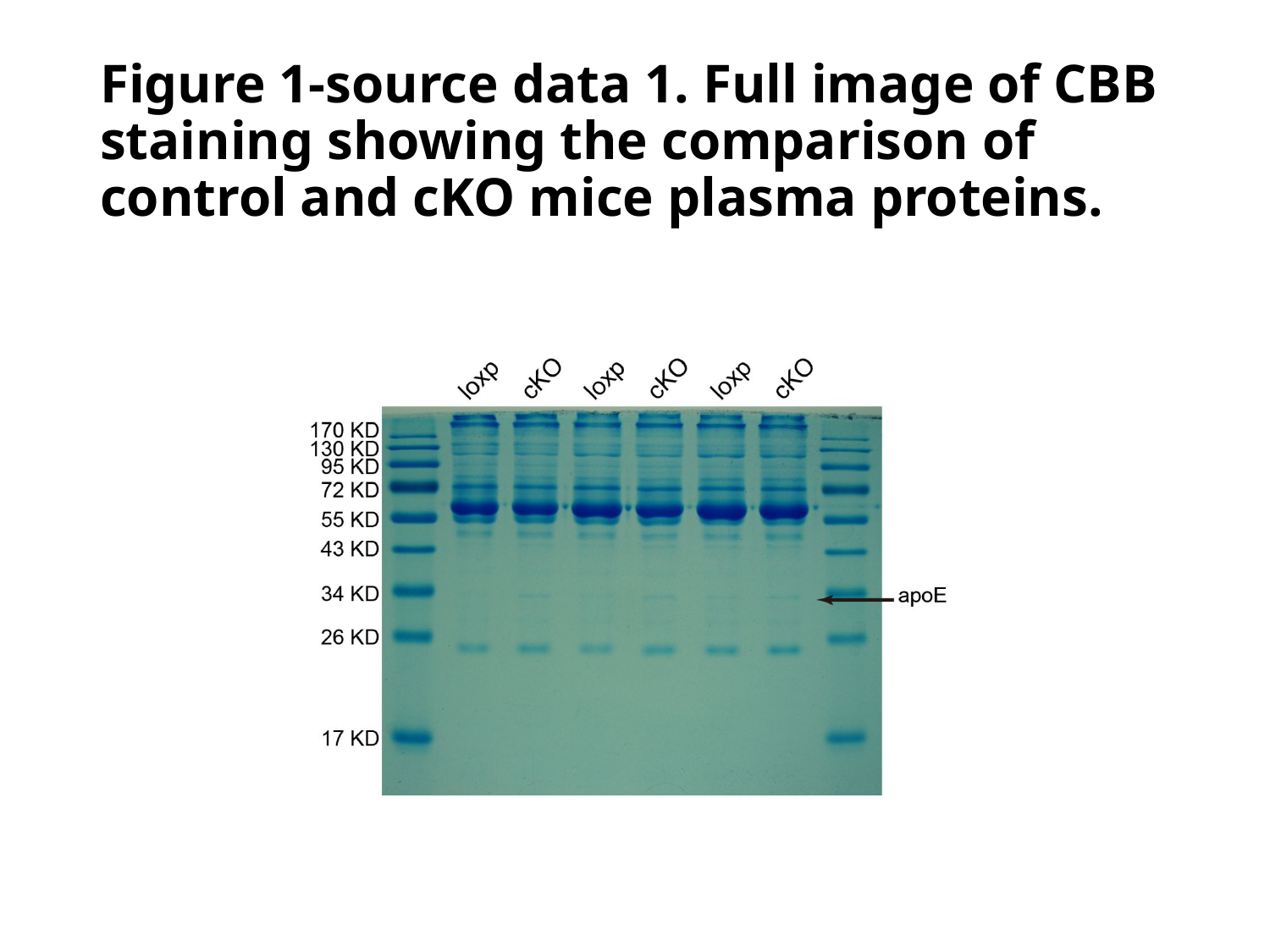

# Figure 1-source data 1. Full image of CBB staining showing the comparison of control and cKO mice plasma proteins.
