# Supplementary material for: BLOS1 mediates kinesin switch during endosomal recycling of LDL receptor
Source: eLife. 2020 Nov 12;9:e58069. doi: 10.7554/eLife.58069 (PMC7688313; doi:10.7554/eLife.58069)
Supplement: Figure 4—source data 1. — (a) Representative confocal image showing the periphery accumulation of LDLR (red) after KIF13A-GFP (green) expression in control primary hepatocytes. (b) Representative confocal image showing the periphery accumulation of LDLR (red) after KIF13A-GFP (green) expression in cKO primary hepatocytes. Magnified insets of boxed areas are shown on bottom. Bar = 10 µm. [file elife-58069-fig4-data1.pptx]

## Slide 1
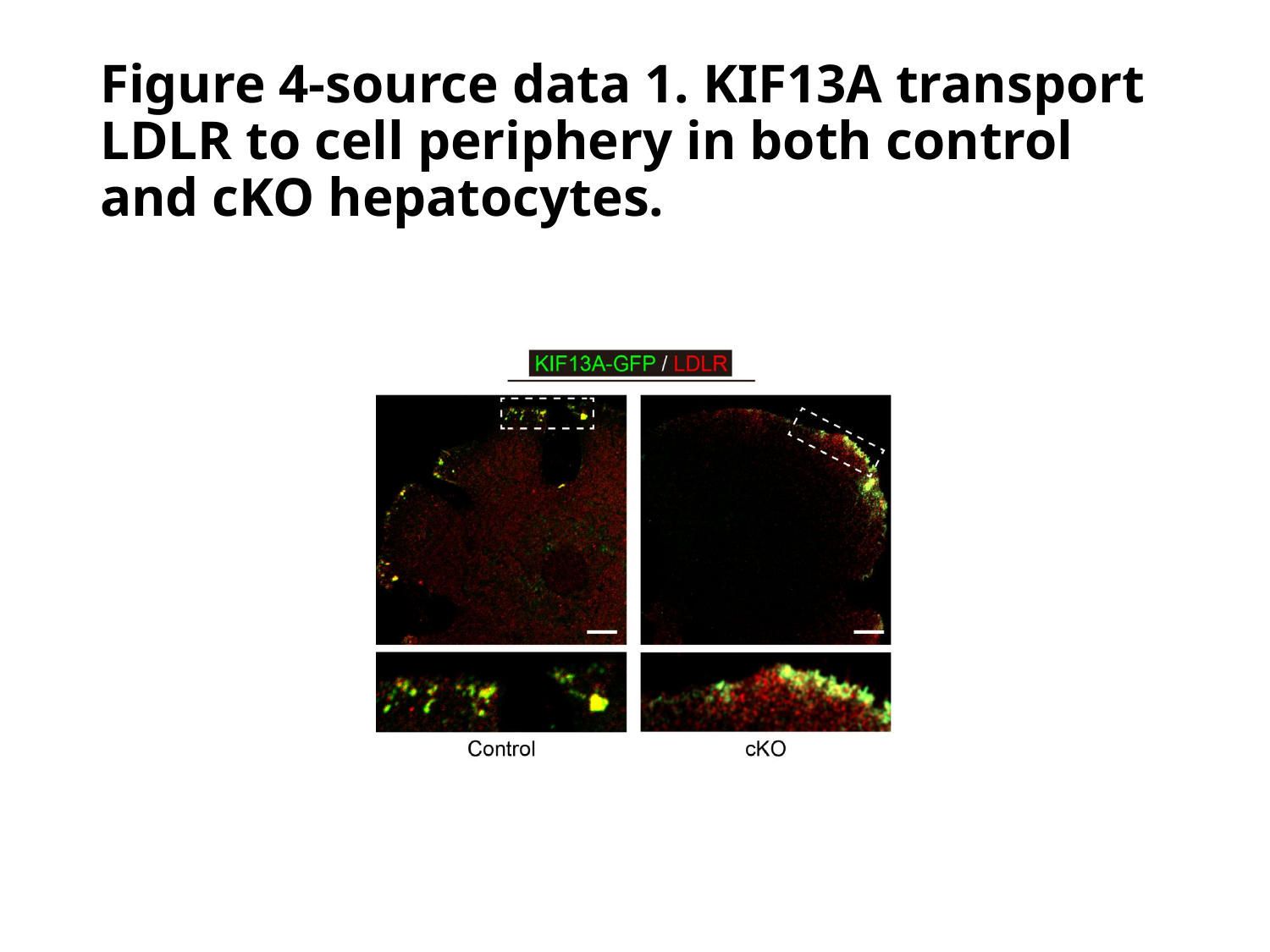

# Figure 4-source data 1. KIF13A transport LDLR to cell periphery in both control and cKO hepatocytes.
