# Supplementary figures and images for: BLOS1 mediates kinesin switch during endosomal recycling of LDL receptor
Source: eLife. 2020 Nov 12;9:e58069. doi: 10.7554/eLife.58069 (PMC7688313; doi:10.7554/eLife.58069)

## Slide 1
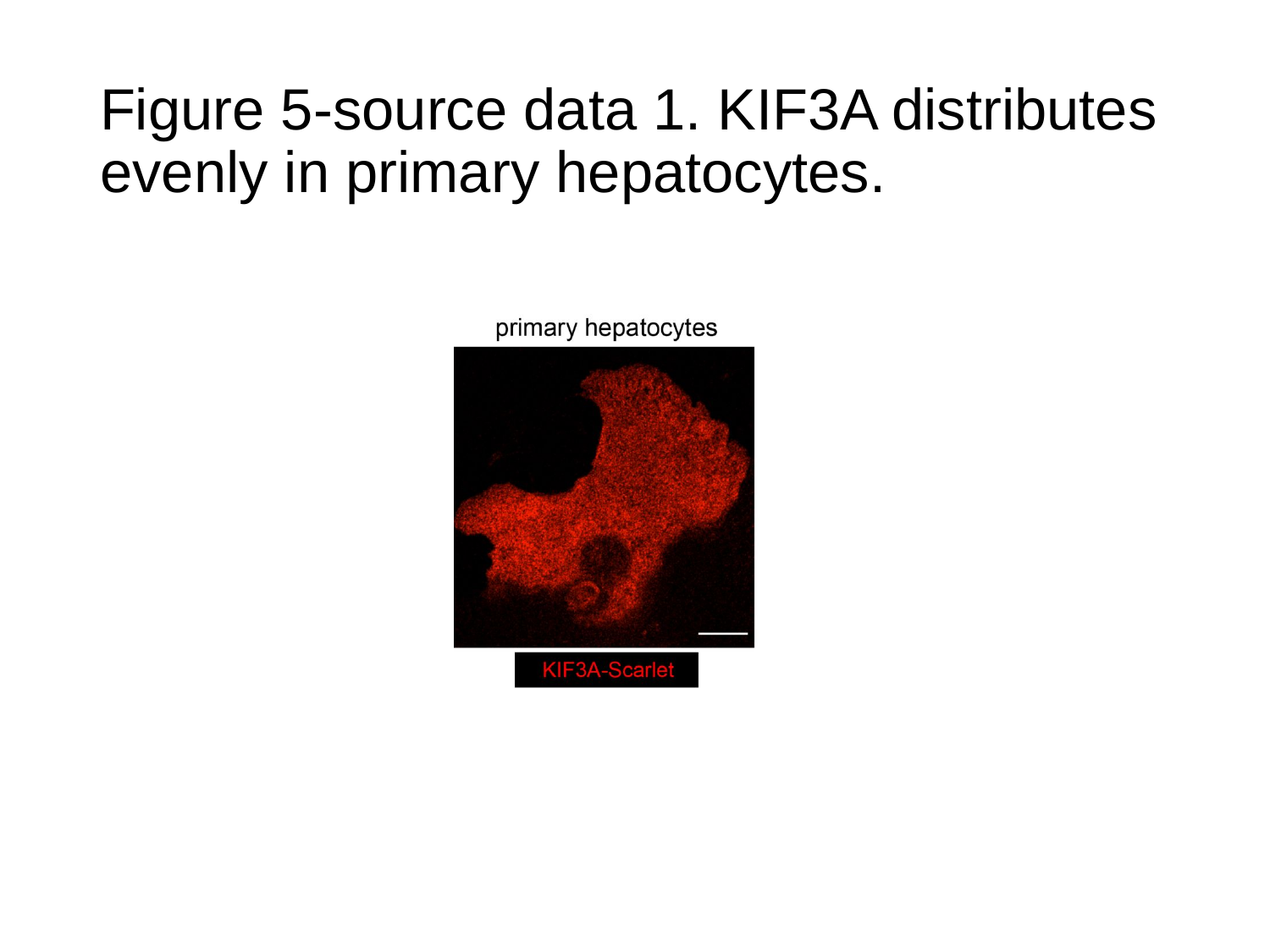

# Figure 5-source data 1. KIF3A distributes evenly in primary hepatocytes.

Supplement: Figure 5—source data 1. — Representative confocal images of non-puncta and even distribution pattern of overexpressed KIF3A-Scarlet (red) in mouse primary hepatocytes. Bar = 10 µm. [file elife-58069-fig5-data1.pptx]
